# Supplementary material for: Intravesical CD74 and CXCR4, macrophage migration inhibitory factor (MIF) receptors, mediate bladder pain
Source: PLoS One. 2021 Aug 23;16(8):e0255975. doi: 10.1371/journal.pone.0255975 (PMC8382170; doi:10.1371/journal.pone.0255975)
Supplement: S1 Table — (PDF) [file pone.0255975.s001.pdf]

S1 Table. Awake Micturition parameters (VSOP; N=6) in all treatment groups.

Intravesical Pretreat.	Intravesical Treat.	Volume (μl)	Frequency (voids/3 hr)
Acute BHA model			
PBS	***Scrambled peptide***	158 ± 13.6	3.7 ± 0.4
PBS	***PAR4-AP***	190 ± 9.3	2.3 ± 0.3
MIF antagonism			
mIgG1	***PAR4-AP***	231 ± 33.6	3.0 ± 0.7
Anti-MIF mAb	***PAR4-AP***	150 ± 15.7	3.5 ± 0.3
CD74 antagonism			
rIgG2b	***PAR4-AP***	177 ± 33.1	4.5 ± 0.7
Anti-CD74 mAb	***PAR4-AP***	118 ± 18.0	5.5 ± 0.5
Vehicle	***PAR4-AP***	167 ± 31.8	2.8 ± 0.3
MIF098	***PAR4-AP***	175 ± 30.7	2.7 ± 0.3
CXCR4 antagonism			
AMD3100	***PAR4-AP***	169 ± 25.5	3.5 ± 0.3
CXCR2 antagonism			
0.1% DMSO	***PAR4-AP***	177 ± 26.3	3.2 ± 0.3
SB225002	***PAR4-AP***	192 ± 62.0	3.5 ± 0.8
