# Supplementary figures and images for: Intravesical CD74 and CXCR4, macrophage migration inhibitory factor (MIF) receptors, mediate bladder pain
Source: PLoS One. 2021 Aug 23;16(8):e0255975. doi: 10.1371/journal.pone.0255975 (PMC8382170; doi:10.1371/journal.pone.0255975)

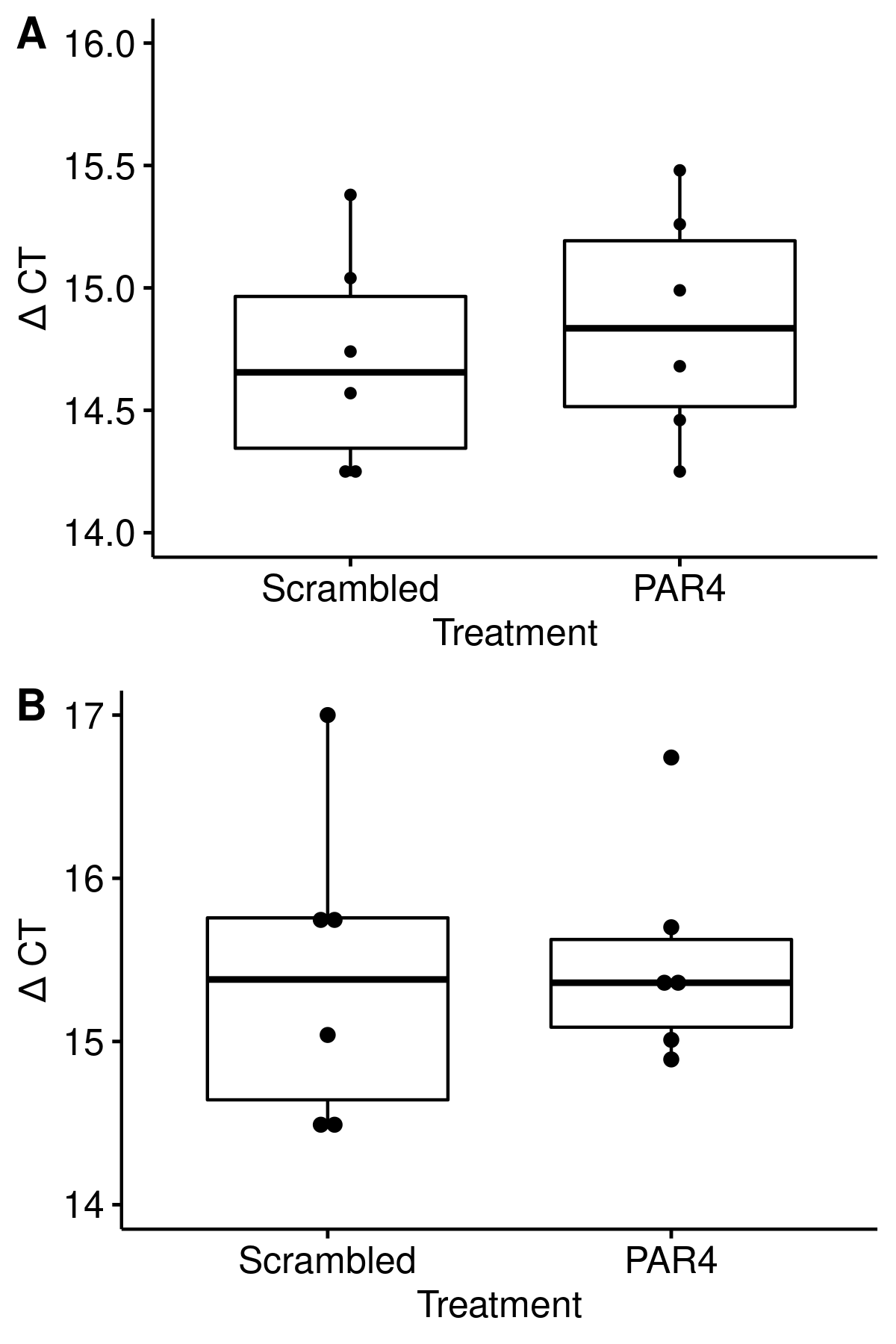

Supplement: S1 Fig — (TIF) [file pone.0255975.s002.tif]

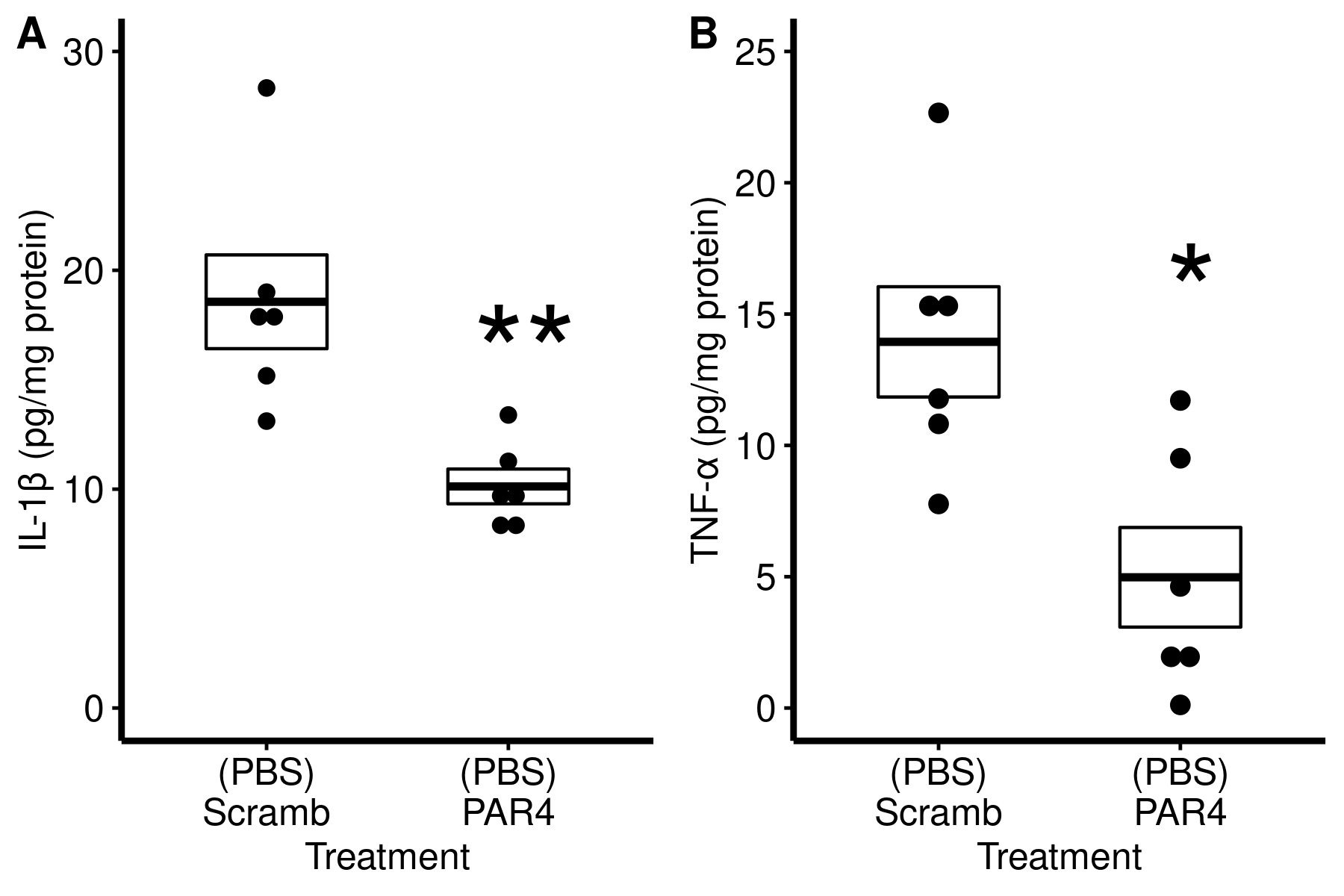

Supplement: S2 Fig — (TIF) [file pone.0255975.s003.tif]
