# Supplementary material for: Intravesical CD74 and CXCR4, macrophage migration inhibitory factor (MIF) receptors, mediate bladder pain
Source: PLoS One. 2021 Aug 23;16(8):e0255975. doi: 10.1371/journal.pone.0255975 (PMC8382170; doi:10.1371/journal.pone.0255975)
Supplement: Attachment — Submitted filename: Response to reviewers_v2.docx [file pone.0255975.s005.docx]

1101 Veterans Drive

Lexington, KY 40502-2236

www.lexington.va.gov

July 6, 2021

Jürgen Bernhagen

Academic Editor

PLOS ONE

Dear Dr. Bernhagen,

We thank the reviewer of our manuscript for the helpful and insightful comments. We addressed the comments as indicated below and consider the manuscript improved. Changes are indicated in the text in the following manner: A) deleted text: crossed out in red: B) New text: in blue with wavy underline.

Changes made in response to Reviewer 1:

1. The title is a bit vague and does not capture the new role for CD74. I would recommend being clear about this as it will get readers attention easier.
   a. **Title was amended to clearly name the MIF receptors**
2. The abstract also should be revised to include this key finding, as well as the in vivo functional observations (which are not clearly articulated).
   a. **Abstract was revised to highlight the role of CD74 and the functional observations have been made clearer.**
3. Given the use of ibudilast (which blocks among other interactions, CD74/MIF) in multiple clinical settings, it would be very useful to use it in these studies. While it may be out of the scope of these studies, this may also be a good use of the CD74 KO mice (developed by one of the co-authors, Dr. Bucala).
   a. **This is an excellent suggestion and we plan to follow up on this in future studies. As this likely represents a separate study we will continue with our current findings having blocked CD74 interactions in two ways: with anti-CD74 mAb and MIF098.**
4. The data in Table 3 are useful, but would be worth presented as a series of graphs. The authors should use their discretion as to how many conditions they show as the rest can be included as supplemental data (in the form of a table).
   a. **Two additional graphs are included:**
      i. **Micturition volume (now Fig 3)**
      ii. **Micturition frequency (now Fig 4)**
   b. **Full table, with all treatment groups is now a supplemental table (Suppl. Table 1)**
5. The immune changes presented are generally qualitative in terms of histology. It would be useful to quantify these, as well as other markers of inflammation via IHC. While it may end up being a negative result (based on the previous Vera laboratory report (PMID 31289792), it would still be worth assessment with appropriate quantification.
   a. **This is a very interesting suggestion and worth following up in future studies. We highlight the quantitative (ELISA) assessments of bladder protein levels of TNF-alpha and IL-1B by adding a supplemental figure (see 5b below). We also extended our discussion and added references to show these are well accepted markers of bladder inflammation (as reported in the literature for other bladder inflammatory models) and we do not see a change in bladder levels of these markers.**
   b. **A figure (Supplemental Fig 2) is presented as supplemental evidence, showing bladder levels of these two inflammatory markers as determined by ELISA.**
6. The qPCR data on IL1-b and TNF-a, while negative, should still be graphed in some manner (as a supplemental figure is sufficient).
   a. **Excellent suggestion. Added as a supplemental figure (Suppl. Fig 1).**
7. In the conclusions, the authors should be more precise about “TLR4” as it is suggested that this is only receptor for HMGB1 (which can bind to other receptors, including TLR2 and RAGE). It may be worth just switching “TLR4” with “HMGB1” receptors or ending the sentence as such: …HMGB1 to result in bladder pain.”
   a. **Concluding statement states “HMGB1 receptors”. We have retained the notion that most likely this is mediated by intravesical TLR4 receptors based on our experimental evidence as cited in the text**.

Changes made in Response to Academic Editor.

1. …please consider creating a summarizing scheme/cartoon that sums up the "induction - vesicle - MIF - receptor - HMGB1 pathway". This would greatly help readers from related fields.
   a. **Thank you for this suggestion. A figure has been added as part of the summary paragraph. (Fig. 7)**

In addition, while animal numbers had been listed through the text and figure captions, they are now also noted in Methods, as requested.

Finally, Funding information was removed from the Acknowledgements section as requested. Funding statement should read:

“This study is funded by NIH (DK121695, PLV; AR049610, RB). The material is the result of work supported with the resources and facilities at the Lexington (Kentucky) VA Health Care System.”

I hope you find these changes acceptable. We look forward to hearing your final decision concerning our manuscript.

Best regards,

Pedro L Vera, PhD.
